# Sleep and the Modern Hypnotics

**Published:** 1909-09

**Authors:** J. M. Fortescue-Brickdale

**Affiliations:** Assistant Physician to the Bristol Royal Infirmary; Lecturer on Pharmacology in the University of Oxford


### SLEEP AND THE MODERN HYPNOTICS.

BY

J. M. FORTESCUE-BRICKDALE, M.A., M.D., Oxon.

*Assistant Physician to the Bristol Royal Infirmary;*
*Lecturer on Pharmacology in the University of Oxford.*

---

ALTHOUGH the phenomena of sleep have been well known for a considerable time, the proximate factors in its causation are still but little understood. The phenomena are: A diminution of metabolic processes all over the body; a relaxation of the voluntary muscles; a diminution in the secretory activity of all glandular structures; a slowing of the heart, and a slowing and deepening of respiration; a fall of blood pressure and temperature; a lessening, and in some cases an alteration, in reflex excitability; a contraction of the sphincter muscle of the iris; and, last but not least, loss of consciousness.

Sleep becomes deeper for about two hours, and then gradually becomes lighter again till the person awakes. Neither falling asleep nor wakening is a sudden event, but only a more or less arbitrarily fixed point in a gradual process.

These phenomena are secondary, and cannot either separately or collectively be regarded as the *cause* of sleep; even the vascular changes, important as they are, are ætiologically inadequate. Ever since Durham's classical experiment, it has been customary to speak of sleep as an "anæmia of the brain"; and though the general opinion is that during sleep there is a marked decrease in the activity of the cerebral circulation, this is not now regarded as the cause of the condition of unconsciousness, but as the result of the fall of blood pressure and the weakened cardiac action.

It might be expected *a priori* that sleep was due to some special condition of the central nervous system, and especially of the cells of the cerebral cortex, and it is therefore peculiarly interesting to find that certain changes have been observed in these structures. Roughly, these changes may be deemed analogous to those seen in a gland cell after its contents have been discharged and it becomes functionally inactive. At the same time, certain physical alterations have been observed in the dendrites which may be of considerable importance, but which are no doubt secondary to processes taking place in the cell itself and in its nucleus. It is, indeed, impossible to imagine that peripheral events, such as the retraction of the dendrites, are primary cell changes.

All that can be said at present, therefore, is that sleeping and waking are the expressions of the rhythmical activity of the central nervous system, and probably correspond to certain anabolic and catabolic changes in the cells of the cerebral cortex.

The subject has been approached from another standpoint by such observers as Meyer and Overton, whose theory as to the mode of action of hypnotic drugs is now well known. They regard the protoplasmic molecule as surrounded by a protective or isolating sheath of fat-like material (such as cholesterin and lecithin) conveniently termed "lipoid." They have observed that the efficacy of various hypnotic drugs is proportional to their solubility in fatty bodies, or rather that the "distribution coefficient" (the ratio between their solubility in lipoids to their solubility in water, $\frac{S_F}{S_W}$) varies inversely as the "liminal value," or the smallest molecular concentration capable of producing a physiological effect.

As the brain is very rich in lipoid substances, Meyer regards this observation as explaining how narcotic drugs gain access to the cerebral cells; and though there is no unanimity of opinion as to the lipoid molecular sheath which Meyer postulates, it is very generally felt that the theory holds good for a certain number of substances, though not for all. For the volatile anæsthetics, Moore and Roaf have shown definitely that an aggregation takes place with the cell proteins which depends on the partial pressure of the gas in the surrounding tissue fluids. It is possibly an absorption phenomenon. All narcotic drugs, whether classed as hypnotics or anæsthetics, must produce their effect by the same means, namely a depression of the function of the cerebral cortical cells. These theories, in so far as they explain anything, only explain the way in which it is possible that these drugs exert their selective influence. This, however, is an extremely important practical point. From the pharmacological point of view, the ideal drug is that which has the greatest power of selection, not necessarily the most physiologically active in a general sense. A drug which is limited in its effects to a comparatively small group of cells is necessarily a remarkably safe therapeutic agent, and can be pressed to produce the full effect required without unpleasant consequences to other cells or organs. A perfect hypnotic should resemble the hypothetical fatigue product, and exert its toxic influence on the cerebral cortical cells only, leaving the lower level centres to pursue their proper functions undisturbed.

Fraenkel has divided hypnotic drugs into three classes, each named after its most typical constituent. The drugs are grouped according to the chemical factor supposed to determine the physiological action. With slight modifications, Fraenkel's classification may be set forth as follows :—

I. **The Alcohol Group.**—In this group the hypnotic action depends on the presence of an alkyl group, especially the ethyl radical $C_2H_5$. It contains four sub-groups :

(i) The alcohols, ethyl alcohol and amylene hydrate.

(ii) The sulphones, sulphonal, trional and tetronal. In these the intensity of the physiological action is least in the first, which contains only two ethyl groups, more marked in the second with three, and most marked in the last with four.

(iii) Substituted amides, urethane and hedonal. These are derivatives of carbamic acid $NH_2,COOH$, in which the hydrogen of the hydroxyl is replaced by ethyl and a radical known as methylpropyl-carbinol respectively. On these substituting alkyls the hypnotic action is thought to depend.

(iv) Closely connected with the last group is that containing the two urea derivatives, veronal and proponal. Urea combined with malonic acid ($COOH,CH_2,COOH$) gives rise to a body known as barbituric acid. The substitution of its two hydrogen atoms by two ethyl or propyl ($CH_3$,$CH_2$,$CH_2$) groups gives rise to the two markedly hypnotic bodies above mentioned.

II. **The Chloral Group** contains a number of bodies dependent for their action on the presence of a halogen, either chlorine or bromine.

(i) From chloral hydrate are derived a number of bodies, of which the best known are chloralose (chloral + glucose) chloralamide (chloral + formamide, H,CO,$NH_2$), dormiol (chloral + amylene hydrate), viferral (a polymer of chloral + pyridine $C_5H_5$,N), somnal (chloral + urethane), trigemin (chloral + pyramidon), hypnal (chloral + antipyrin), hypnone (chloral + acetophenone).

(ii) Isopral is trichlor-isopropyl-alcohol.

(iii) Chloretone is tertiary trichlor-butyl-alcohol.

The bromine derivatives are :—

(i) Neuronal; or acetamide $CH_3$CO,$NH_2$, in which the three hydrogens are replaced by two ethyl groups and a bromine atom.

(ii) Bromural; a urea derivative containing one atom of bromine + a valerianyl group $(CH_3)_2$CH,Br,CO – NH,CO,$NH_2$.

(iii) Brometone; the corresponding derivative to chloretone.

III. **The Paraldehyde Group,** which contains bodies which are hypnotic by virtue of their being aldehydes (paraldehyde, a polymer of acetaldehyde $CH_3$,CHO), or ketones (propion, diethylketone $C_2H_5$,CO,$C_2H_5$; and hypnone or acetophenone, phenylmethyl-ketone $C_6H_5$,CO,$CH_3$).

It will at once be apparent that whatever else the synthetic chemists have omitted to do, they have not failed to provide the physician with a large choice in the matter of hypnotic drugs. The choice is, indeed, so large that it is impossible to deal fully with all these bodies within the limits of this article. Moreover, although Fraenkel's classification is most interesting from a scientific point of view, it is not of any great value therapeutically, so that for practical purposes another grouping may be adopted :—

*Group I.*—Hypnotic drugs presenting definite disadvantages.

(1) **Tetronal.** This drug, which is best given in cachets, is too dangerous for ordinary use. The dose is 5 to 20 grains.

(2) **Isopral** is a powerful hypnotic, but probably owing to the presence of a chlorine atom has a depressant action on the heart, which renders it an undesirable drug. It also occasionally upsets the digestion. Its action is prompt (ten to twenty minutes), and the sleep produced is light and lasts for from five to ten hours. It does not generally act in presence of pain. The usual dose is 5 to 8 grains, but 15 or even 30 may be given. It is somewhat insoluble, and is best given in sugar-coated dragées.

(3) **Chloralose** differs from other chloral compounds in exaggerating the reflexes and eventually producing convulsions. It is seldom given owing to the fact that it may cause tremor and excitement, possibly owing to the formation of a second body, parachloralose, which has no hypnotic action. The dose is 2 to 8 grains, usually in cachets owing to the disagreeable taste.

(4) **Chloral urethane** (somnal, ural, or uralium) has a marked toxic action, which is more powerful and lasts longer than its narcotic effect. Somnal is an alcoholic solution, and is given in doses of 15 to 30 minims.

*Group II.*—Hypnotic drugs possessing no special advantages over others more generally employed.

(1) Trigemin, hypnal, hypnone, which are merely addition products. (2) Brometone, the dose of which is 30 grains, has no special advantages. (3) Propion and hypnone: the former is efficient, but unpleasant to take, and the latter is but slightly hypnotic, though not nasty.

*Group III.*—The mild hypnotics. These are urethane, hedonal, amylene hydrate, chloretone, chloralamide and viferral.

(1) **Urethane.**—A white powder, soluble in water, and almost tasteless. Is too mild in its action to be of much use except for children, to whom 1½ grains may be given for every year up to 15. Tolerance is soon established.

(2) **Hedonal** is more powerful. It is also in the form of a white powder, but is somewhat insoluble, and has an unpleasant burning taste. It must, therefore, be given in cachets, or dissolved in spirit and mixed with cinnamon as a flavouring agent. The dose is 7½ to 15 grains, but an adult male often requires as much as 30 grains. Sleep should be produced in about half an hour, and lasts seven hours or so. It has no depressant action on the heart.

(3) **Amylene hydrate** is a colourless liquid with a burning taste and a characteristic aromatic odour. It is very hygroscopic, and must be kept protected from light. The dose is from 40 to 60 minims. It seldom gives rise to any serious toxic symptoms, but minor disturbances are not uncommon. It is usually prompt in action (fifteen to twenty minutes), but occasionally there is considerable delay. Six hours' sleep should be produced. A preliminary stage of excitement sometimes occurs. It is apparently nearly completely oxidised in the body, like other alcohols. Tolerance is established in about a week. Its hypnotic power has been estimated at about half that of chloral hydrate.

(4) **Chloralamide** is a white insoluble powder, which should be prescribed dissolved in warm spirit one hour before its action is required. The dose is 20 to 30 grains. It is a remarkably safe hypnotic, as its action depends on its decomposition in the body (chloral being formed), and this only takes place very slowly. It should never be given in a cachet, as its action is then often delayed for twelve or fourteen hours.

(5) **Chloretone** is a somewhat insoluble substance, and is best prescribed in petroleum emulsion. The dose is 5 to 15 grains. It is not a cardiac depressant in ordinary doses, and seldom upsets the stomach. It has, however, only very slight hypnotic action.

(6) **Viferral** is a white powder, with a bitter taste, easily soluble in hot water. The taste may be disguised by Syr. Limonis, or it may be given in a tablet or cachet. It is not decomposed in the stomach. The minimum dose is 15 grains, but 20 or 30 grains are often required. It appears to be a mild hypnotic, but there is at present but little evidence as to its value.

*Group IV.*—The hypnotics of medium strength are paraldehyde, sulphonal and bromural.

(1) **Paraldehyde** is a colourless fluid, the very unpleasant odour and taste of which it is practically impossible to conceal. It should, therefore, be given in cachets. The dose is ½ to 2 drachms.

It produces a light sleep of from five to eight hours' duration. It has no depressant action on the heart, and seldom produces digestive disturbances. It is usually very reliable in its action.

(2) **Sulphonal** is a somewhat insoluble powder, the dose of which is 10 to 30 grains. It may be dissolved in hot tea or water, but should not be given in tablets. Sleep is not produced for about two hours, and is apt to be prolonged over the next day. It has no action on the heart, but is a blood poison, producing hæmatoporphyrin in the urine. Its disadvantage is that it is slow and uncertain in action, and that the toxic dose varies enormously in different individuals. It may show cumulative action.

(3) **Bromural** consists of white, slightly bitter crystals, with a camphoraceous odour, soluble in hot water, spirit or alkalies. It should not be given in tablets, but should be crushed and dissolved. The dose is 5 to 15 grains. It produces two to five hours' sleep, usually in twenty to twenty-five minutes, but may act more promptly. It sometimes gives rise to headaches, even in moderate doses, and sometimes produces an irritable drug rash. It is well borne by children. The sleep obtained by its means is very quiet and natural, but it cannot be relied upon in severe cases of insomnia, or where pain is present.

*Group V* contains the most powerful hypnotic drugs, namely veronal, proponal, chloral hydrate, neuronal and trional.

(1) **Chloral hydrate** has been employed for forty years, and may be taken as the standard hypnotic. Some recent writers entirely condemn it, but there seems no reason for this extreme view. Its disadvantages and its value are so well known that there is no need to consider them in an article like the present one. It may, however, be remarked that it might be more frequently employed at the present time, to the exclusion of some newer and less reliable drugs.

(2) **Veronal** is a white crystalline powder, slightly bitter, and possessing no odour. It is only slightly soluble in water (1 to 145 at 60° F.). It should *never* be given in solid form, as its action is then uncertain and delayed. It is fairly easily dissolved in hot fluids (1 to 12 boiling water), and may be given in tea, soup, &c.

Milk should not be employed, as a bitter alkaline decomposition product is formed (Von Leyden). The necessary dose is still somewhat indeterminate: 3 to 5 grains appears to be the ordinary minimum, but this has often to be exceeded in asylum practice. Larger doses than 15 grains usually produce unpleasant after-effects—drowsiness, headache, nausea, chilliness and irregular pulse. Occasionally constipation occurs. Cases of severe and fatal poisoning have been reported, but these are rare. Its insolubility prevents rapid absorption, and large doses have sometimes been taken without further result than prolonged sleep. It has no bad action on heart, respiration, or kidneys, and is slightly diuretic. Sleep is usually induced in fifteen to thirty minutes, and lasts from six to eight hours. There is some evidence that veronal has a cumulative action, but on the other hand, when given continuously, tolerance is established in some patients, who are then also unaffected by trional (Ziehen). **Sodium veronal** is similar in action, and is given in the same doses. It has the advantage of being more soluble, and may thus be conveniently given in combination with other drugs and *per rectum.*

(3) **Proponal** is a colourless crystalline body, very insoluble in cold water, but easily so in alkalies. The solutions have a slightly bitter taste. It is an even more powerful hypnotic than veronal, and is effective in half the doses usually given of the latter drug. Sleep is produced in fifteen minutes, and is often prolonged. It is thus more prompt in action than veronal, but the toxic dose is but little above the hypnotic—7½ grains borders on the dangerous. Tolerance is rather rapidly established in some cases (three or four days).

(4) **Neuronal** is a colourless crystalline powder. It is but slightly soluble in cold water, but easily so in alcohol. It has a slightly camphoraceous odour and a bitter taste. It should not be given in an alkaline medium, as under these circumstances prussic acid is formed. The dose is 5 to 15 grains, but in severe cases, or when pain is present, larger doses (up to 30 grains) are necessary. Sleep is produced in 20 to 30 minutes, and lasts seven to eight hours. It not infrequently fails to produce its effect, and must, therefore, be regarded as uncertain, though powerful. It is also somewhat depressant in its after-effects (it contains 41 per cent. bromine), and is rather apt to produce digestive disturbances.

(5) **Trional** is a colourless, somewhat insoluble powder. It should be given dissolved in spirit, or as a powder in cachets, but never in tablets. The solutions leave a bitter taste. It is usually more prompt in its action than sulphonal, and does not produce drowsiness on the following day. Smaller doses, moreover, are effective. On the whole, it is safe and reliable ; the toxic symptoms are similar to those produced by sulphonal. A useful combination contains 15 grains of trional and 30 minims of paraldehyde, given in mist. amygdalæ (B.P.). It is said to act four or five times more powerfully than trional alone, and to avoid the delay in hypnotic effect which sometimes occurs. Trional is useless where pain is present.

